# Novel Alzheimer’s disease-associated variants and genetic interactions identified from UK biobank whole-exome sequencing data using IBI-DT

**DOI:** 10.1038/s41598-026-58159-w

**Published:** 2026-06-26

**Authors:** Jin Ren, Rui Yin, Jinying Zhao, Jinling Liu

**Affiliations:** 1 Department of Epidemiology, University of Florida, Gainesville, FL 32611 USA; 2 Department of Health Outcomes and Biomedical Informatics, University of Florida, Gainesville, FL 32611 USA; 3 Health Informatics Institute, University of South Florida, Tampa, FL 33620 USA

**Keywords:** Alzheimer’s disease, Genome-wide association study, Individualized Bayesian inference-decision tree, Whole exome sequencing, Gene-gene interactions, AD prediction, Computational biology and bioinformatics, Genetics, Neuroscience

## Abstract

Alzheimer’s disease (AD) is a highly heritable neurodegenerative disorder whose genetic architecture remains incompletely understood, particularly with respect to rare variants and higher-order interactions. We applied Individualized Bayesian Inference-Decision Tree (IBI-DT) to 790,000 whole-exome sequencing variants from 8,292 unrelated White British individuals in the UK Biobank, using genome-wide association analysis (GWAS) based on Fisher’s exact test as a marginal association comparator and evaluated the findings in an independent ADSP-Discovery ICE cohort of 1,560 unrelated individuals with 852,997 variants. In the discovery cohort, IBI-DT identified 178 significant variants using empirical null calibration, which mapped to 173 genes, whereas GWAS identified 16 significant variants that mapped to four genes. Compared with GWAS, IBI-DT prioritized more rare variants among the top 178 signals (147 vs. 66 with minor allele frequency ≤ 0.01) and identified variants with less correlated, less redundant linkage patterns. The IBI-DT-prioritized variants included established AD genes as well as additional biologically supported genes not prioritized by GWAS. Replication supported three variants and four genes across cohorts, including gene-level replication of *KIF14* and *ZNF90*, which were replicated only by IBI-DT. IBI-DT also identified significant gene-gene and gene-environment interactions and biologically plausible enriched pathways. Neural networks trained on IBI-DT-prioritized variants outperformed GWAS-based models (AUC 0.67 vs. 0.63). Overall, these findings indicate that IBI-DT complements GWAS by recovering established AD signals while prioritizing rare, less redundant, and interaction-related AD-associated signals.

**Supplementary Information:**

The online version contains supplementary material available at https://doi.org/10.1038/s41598-026-58159-w.

## Introduction

Alzheimer’s disease (AD) is the most common form of dementia and a leading cause of disability among older adults^1,2^. Twin and family studies estimate the heritability of AD ranges from 60% to 80%^3,4^, indicating a substantial genetic contribution to disease risk. Among known genetic factors, the ε4 allele of *APOE* represents the strongest and most consistently replicated risk variant^5^; however, it accounts for only a fraction of the observed heritability. This gap between estimated heritability and explained genetic risk suggests that additional genetic mechanisms underlying AD remain to be elucidated.

Over the past four decades, genome-wide association studies (GWAS) have substantially advanced the understanding of AD genetics by identifying more than 40 loci associated with late-onset AD^6–10^. Recent large-scale GWAS meta-analyses have further expanded this landscape by dramatically increasing sample size and statistical power. For example, Wightman et al. analyzed over 1.1 million individuals across multiple cohorts and identified 38 independent AD-associated loci^11^, while Bellenguez et al. examined more than 780,000 individuals and reported 75 risk loci, including 42 novel associations^12^. These studies demonstrate that AD GWAS has reached an unprecedented scale and maturity. Nevertheless, the majority of identified variants exhibit modest marginal effects at the population level, and even the largest GWAS to date explain only a limited proportion of AD heritability, indicating that substantial genetic contributions remain unaccounted for.

Several methodological challenges inherent to standard GWAS frameworks contribute to this missing heritability. GWAS are designed to detect marginal associations averaged across large, genetically heterogeneous populations, which limits their ability to capture variants whose effects are context-dependent or confined to specific genetic or environmental backgrounds^13,14^. As a result, variants with small or moderate effects, especially the rare variants, may be diluted when analyzed at the population level. In addition, systematic discovery of gene-gene and gene-environment interactions remain challenging due to the high dimensionality of the search space and the substantial computational burden required for interaction testing at genome scale^15,16^. Together, these limitations restrict the ability of GWAS to fully characterize the complex and heterogeneous genetic architecture of AD.

These challenges highlight the need for complementary analytical approaches that move beyond population-level marginal association testing. In this study, we applied the Individualized Bayesian Inference-Decision Tree framework (IBI-DT)^17^ to AD genetic analysis. Unlike traditional GWAS, IBI-DT models genetic effects within more homogeneous genotype-defined subgroups using a decision tree structure guided by Bayesian marginal likelihood. This design enables IBI-DT to prioritize both common and rare variants and to identify higher-order gene-gene and gene-environment interactions^17^. IBI-DT provides a complementary framework to conventional population-level association approaches, as well as regression-based interaction analysis and rare-variant burden methods. Unlike burden methods, which aggregate multiple variants within a gene or region, IBI-DT operates at the single-variant level and prioritizes variant-specific and interaction-related signals within subgroup-defined contexts. Thus, IBI-DT is designed to reveal subgroup-structured and interaction-related signals that may be weakly captured by marginal association or aggregate burden tests. The framework has previously been applied to cancer driver and interaction discovery^17^. Whereas the prior cancer application focused primarily on gene-level driver and interaction discovery, the present study applies IBI-DT directly at the variant level in AD WES data, allowing us to evaluate both single-variant prioritization and variant-level interaction structures. In this study, we used single-variant GWAS based on Fisher’s exact test as a simple marginal association comparator to evaluate how IBI-DT differs from conventional population-level single-variant testing. By explicitly accounting for genetic heterogeneity at the individual and subgroup levels, IBI-DT provides a complementary perspective on AD genetic architecture and may help identify variant and interaction signals that may be overlooked by conventional GWAS methodologies.

## Methods

### Discovery cohort and replication cohort

The discovery cohort included the Whole Exome Sequencing (WES) data from the UK Biobank (UKB, https://www.ukbiobank.ac.uk/)^18^, a prospective cohort of over 500,000 individuals, enrolled at ages from 40 to 69 with genetic and phenotypic data. We selected unrelated participants of white British ancestry with concordant reported and genetic sex, no sex chromosome aneuploidy, no heterozygosity outliers, and available WES data. Participants lacking parental age or illness information, required for AD-by-proxy calculation, were excluded. AD-by-proxy has been shown to have a genetic correlation with AD and has been widely used in numerous AD GWAS studies^19,20^. We defined strict AD cases as individuals with clinically confirmed AD. Controls were individuals without AD and without any AD-proxy status (e.g., parental AD), because proxy AD does not represent true AD and inflates genetic risk relative to true controls.

Initially, we had 1,728 cases and 199,747 controls. To address the extreme imbalance in the case-control ratio, we performed an individually matched nested case-control (NCC) study, matching each case to five controls by age at enrollment (Data-Field 21022; ± 2 years) and sex (Data-Field 22001) from 199,747 controls. Each control sample was used only once. This yielded 10,413 participants with 1,728 AD cases and 8,685 non-AD controls. From this matched population (10,413 participants), we then randomly selected 80% of 1,728 cases and matched each with 5 controls using the same age and sex criteria, forming a final discovery cohort of 8,292 individuals (1,382 cases and 6,910 controls) for GWAS and IBI-DT analyses. The remaining 2,121 samples (346 cases and 1,775 controls) were designated as the test dataset for evaluating the AD prediction model. The average age at enrollment of the discovery cohort was 63.6 years (standard deviation: 4.3), with 53.1% female participants. Among AD cases in the discovery cohort, the mean estimated age at first AD diagnosis was 75.1 years (standard deviation: 5.3). The estimated age at first AD diagnosis was based on the earliest AD-related inpatient diagnosis. This was determined by linking AD-related ICD-10 codes (Data-Field 41270) to corresponding diagnosis dates (Data-Field 41280) and then calculated age using year of birth (Data-Field 34). In the matched analytic sample of 8,292 individuals, we examined *APOE* ε4 genotype status using rs429358 and rs7412. *APOE* ε4/ε4 homozygotes were defined as rs429358 = CC and rs7412 = CC. Heterozygous ε4 carriers included ε3/ε4, defined as rs429358 = CT and rs7412 = CC, and inferred ε2/ε4, defined as rs429358 = CT and rs7412 = CT following standard *APOE* two-SNP coding practice. Because phased haplotypes were not available, the double-heterozygous genotype was classified as ε2/ε4 while treating the alternative rare noncanonical haplotype configuration as negligible. In total, 320 individuals were classified as ε4/ε4 homozygotes, corresponding to 3.86% of the matched analytic sample, and 2,367 were classified as heterozygous ε4 carriers, including 2,193 ε3/ε4 and 174 inferred ε2/ε4 individuals, corresponding to 28.55%.The independent replication cohort used WES data from the Alzheimer’s Disease Sequencing Project (ADSP) project (NG00067.v13, “R2 20K WES Project Level VCF) via NIA Genetics of Alzheimer’s Disease Data Storage Site (NIAGADS). The replication analysis focused on the ADSP Discovery Illumina Rapid Capture Exome (ICE) kit sub-study. To ensure consistency with the discovery cohort, we excluded replicate samples (intended or unintended), related individuals, and non-White participants using metadata from the Genome Center for Alzheimer’s Disease (GCAD). After applying these exclusions and additional quality control, the replication cohort consisted of 1,560 unrelated White individuals, including 1,070 AD cases and 490 controls. The average age in this cohort was 81.4 years (standard deviation: 7.5), with 52.3% being female (Supplementary Table S1).

We performed a single-variant case-control power calculation using the R package genpwr^21^ to evaluate detectable effect sizes in the discovery cohort. The calculation used 1,382 AD cases, 6,910 controls, an exome-wide Bonferroni-corrected significance threshold of (0.05/790000 = 6.33 × 10^-8), and a dominant genetic model. Across representative MAF values of 0.005, 0.01, 0.05, 0.10, and 0.20, the discovery cohort had approximately 80% power to detect odds ratios of 4.36, 2.98, 1.76, 1.56, and 1.46, respectively. These calculations indicate that the discovery cohort was well powered to detect common variants with moderate effects but had limited power to detect rare variants with modest effects using standard marginal association testing.

### AD Phenotype definition and non-genetic factors

We extracted ICD-10 codes from the UK Biobank as AD phenotypes in the discovery cohort. Participants diagnosed with G30.0, G30.1, G30.8, G30.9, F00.0, F00.1, F00.2, or F00.9 were classified as AD cases. AD-by-proxy^19^ was determined based on participants’ AD status, their parents’ disease status, and age at death, and individuals without AD or AD-by-proxy as controls.

Six non-genetic factors were included in the analysis (see Supplementary Table S2) and converted to binary variables: length of mobile phone use was based on the question “For approximately how many years have you been using a mobile phone at least once per week to make or receive calls?” (≥ 8 years = 1, < 8 = 0; Data-Field 1110). Insomnia was derived from the question “Do you have trouble falling asleep at night or do you wake up in the middle of the night?” (usually = 1, not usually = 0; Data-Field 1200). Educational qualification was based on the question “Which of the following qualifications do you have?” (without any certification = 1, with at least one certification = 0; Data-Field 6138). Time spent watching TV was obtained from the question “In a typical DAY, how many hours do you spend watching TV?” (≥ 4 h/day [highest 25%] = 1, < 4 = 0; Data-Field 1070). Age (≥ 65 = 1, < 65 = 0; Data-Field 21022), and Townsend deprivation index ( > − 3.81 [lowest 25%] = 1, and ≤ − 3.81 = 0; Data-Field 22189).

In the replication cohort, the ADSP-ICE sub-study, cases were individuals with either prevalent or incident AD. Controls were participants without prevalent, incident AD, or mild cognitive impairment (MCI). Participants with missing AD status, other forms of dementia, or MCI were excluded from the analysis.

### Genotypes and quality control

We utilized the final release of UKB WES data. Initially, we had 26,388,327 single-nucleotide polymorphisms (SNPs) or variants and 469,835 individuals. Quality control was performed using PLINK2 with the following thresholds: minor allele frequency (MAF) ≥ 0.0001, mind ≤ 0.01, geno ≤ 0.01, HWE ≤ 1e-6, max-alleles = 2, and SNPs-only. Missing genotypes were imputed using the mode, and SNPs were encoded using a dominant model. After filtering, 790,000 SNPs and 10,413 samples (1,728 AD cases and 8,685 controls) were retained.

For the replication cohort, low-quality variants were filtered using GCAD-provided flags, which were generated separately for each sub-study. Variants were excluded if the VFLAGS was not “PASS” (when the GATK “FILTER” ≠ “PASS” or when the tranche ≥ 99.7% or all genotypes have DP < 10 and GQ < 2, or monomorphic, or call rate ≤ 80%, or average mean depth > 500 reads, or outside of exome target capture region ± 7 bp). Additionally, variants with allele balance in heterozygous genotypes (ABHet; ratio of reference reads to total reads at heterozygous sites) < 0.25 or ABHet > 0.75, or those with a call rate < 95% were excluded. After filtering, 852,997 variants were retained.

### Genome-wide association study

We conducted GWAS analyses on autosomal variants of the AD phenotype. A dominant model was implemented to generate 2 × 2 contingency tables, where homozygous reference alleles were coded as 0, and homozygous alternative and heterozygous alleles were recorded as 1. GWAS was performed using Fisher’s Exact Test in both the discovery cohort and replication cohorts. The test was conducted using the “fisher_exact” function from Python (v3.10.0) scipy. stats library.

### Individualized Bayesian-Inference Decision Tree (IBI-DT)

Individualized Bayesian-Inference Decision Tree (IBI-DT) is a Bayesian framework that integrates Individualized Bayesian Inference (IBI) with a marginal-likelihood-guided decision tree to identify disease-associated variants and higher-order gene-gene and gene-environment interactions under genetic heterogeneity. The IBI framework was originally developed to perform Bayesian inference within genotype-defined subgroups in order to address population heterogeneity and improve detection of variants with context-specific effects. IBI-DT extends this framework by introducing a recursive decision tree structure that enables systematic discovery of variant interactions while preserving individualized and subgroup-level inference^17,22^.

IBI-DT constructs decision trees using Bayesian marginal likelihood as the splitting criterion at each node. At each split, candidate variants are evaluated based on their Bayesian Dirichlet equivalent uniform (BDeu) marginal likelihood score, and the variant that maximizes the marginal likelihood is selected. A depth-dependent prior penalty is imposed on tree growth to discourage overfitting. The algorithm includes two hyperparameters: a pruning parameter * q*, which controls tree complexity, and the number of bootstrap trees * k*. The parameter * q* controls the structure prior that penalizes deeper trees: smaller * q* values impose stronger regularization and support shallower trees, whereas values closer to 1 allow deeper and more complex trees. Specifically, the tree-structure prior is defined as $\:P\left(\mathrm{Model}\right)={q}^{\mid\:\mathrm{path}\mid\:}$, where $\:\mid\:\mathrm{path}\mid\:$ is the tree level or path length of the current node. The score for each node includes the term $\:\mathrm{log}\left[P\left(\mathrm{Model}\right)\right]=\mid\:\mathrm{path}\mid\:\mathrm{*}\mathrm{l}\mathrm{o}\mathrm{g}q$, and Since 0 < * q* <1, $\:\mathrm{l}\mathrm{o}\mathrm{g}q$ < 0, deeper nodes receive increasingly negative penalties. As a result, a split is retained only when the improvement in Bayesian marginal likelihood is sufficient to overcome the depth penalty. This regularization helps constrain greedy tree growth and reduce overfitting in the presence of many variants. In addition, because each split is selected conditional on variants already selected along the tree path, highly correlated variants that provide redundant information are less likely to be selected again unless they improve the marginal likelihood within a downstream subgroup.

In this study, * q* was set to 0.6 and * k* to 200, consistent with prior IBI-DT applications^17^. Sensitivity analyses were performed to evaluate how * q* affected tree complexity and search breadth in the AD dataset. Across representative values * q* = 0.2, 0.4, 0.6, and 0.8, the corresponding average tree depths were 11, 16, 24, and 42, and the numbers of unique variants appearing across 200 trees were 5,898, 10,975, 16,397, and 22,718, respectively. These results show the expected tradeoff between overly restricted exploration at lower * q* values and increased model complexity at higher q values. Based on this sensitivity analysis, * q* = 0.6 provided a practical balance between search breadth and tree complexity in the AD dataset. The parameter * k* = 200 denotes the number of bootstrapped IBI-DT trees and was selected to improve the stability of aggregated variant and interaction signals across resampled datasets while maintaining feasible computation.

To assess single-variant importance, IBI-DT employs a bootstrap ensemble strategy. Across the $\:k\:$= 200 bootstrapped trees, each variant’s importance was quantified by the number of times it appeared as a splitting variable, denoted by $\:\alpha\:$. Variants with higher $\:\alpha\:$ values are interpreted as having stronger and more stable associations with the phenotype across heterogeneous subgroups. For interaction analysis, variant-variant interactions were identified from parent-child relationships within the tree structures. Interaction importance was quantified by counting the frequency with which a given variant pair co-occurred across trees, denoted by $\:\beta\:$, with higher $\:\beta\:$ values indicating stronger evidence of interaction. Conceptually, $\:\alpha\:$ and $\:\beta\:$ serves as a computationally efficient approximation to Bayesian model averaging, because rather than summing over all possible tree structures and variant combinations, IBI-DT bootstraps the data, fits trees under a Bayesian criterion, and tallies each variant’s recurrence across the ensemble. We previously explored more formal BMA-related alternatives, including importance-sampling-based posterior model weighting, but these approaches were less robust or substantially more computationally intensive in practice. Therefore, in the current implementation, we retained $\:\alpha\:$ and $\:\beta\:$ as empirical ensemble-stability measures and calibrated their significance using random-node experiments described below.

IBI-DT models two biologically interpretable types of interactions. Conjunctive interactions occur when both the parent and child variants are mutated (genotype status “1”), jointly influencing downstream molecular or phenotypic outcomes. Disjunctive interactions occur when the parent variant is unmutated (genotype status “0”), indicating that the child variant may exert regulatory influence in the absence of the parent variant, consistent with compensatory or alternative pathway effects^17^.

To assess statistical significance of identified variants and interactions, a random-node control experiment was performed. For each of the 200 bootstrapped IBI-DT tree structures, the splitting variants at all nodes were replaced with randomly selected variants drawn from the full variant pool, while preserving the original tree topology. This process generated 200 randomized trees per experiment. Variant importance ($\:\alpha\:$) and interaction importance ($\:\beta\:$) were recalculated for each randomized ensemble. The entire procedure was repeated 200 times to generate empirical null distributions for $\:\alpha\:$ and $\:\beta\:$. The maximum values observed in these null distributions, denoted $\:\alpha\:$' and $\:\beta\:$', were used as significance thresholds. Variants with $\:\alpha\:$ > $\:\alpha\:$' and interactions with $\:\beta\:$> $\:\beta\:$' were considered statistically significant, indicating evidence beyond random tree structure effects.

Importantly, IBI-DT remains computationally efficient for high-order interaction inference by combining closed-form marginal likelihood calculation, vectorized and parallel matrix computation, GPU acceleration, and a greedy tree-based search guided by the observed data. In this study, genome-wide analysis of 790,000 variants in 8,292 subjects using an NVIDIA B200 GPU was completed in approximately three hours for the primary setting of * q* = 0.6 and *k* = 200, enabling high-order interaction discovery.

For practical application to other datasets, we recommend using prior parameter settings as starting points, performing dataset-specific sensitivity analyses for * q* and the number of bootstrapped trees, reporting empirical null distributions for $\:\alpha\:$ and $\:\beta\:$, and interpreting clustered regional signals in the context of LD. These steps help distinguish stable IBI-DT signals from patterns driven by tree instability or excessive model complexity.

### Gene annotation with ANNOVAR and AD gene reference lists

Statistically significant variants or variant-variant interactions were mapped to their corresponding genes to enable interpretation at the gene level, facilitating downstream functional and pathway analyses.

The WES data from both the discovery and replication cohorts were mapped to GRCh38. Gene annotation was performed with ANNOVAR^23^. Each variant was assigned to its nearest gene and classified by predicted functional impact. Identified genes were compared to two reference lists: the ADSP-Lancet AD genes list and the Multi-Platform AD genes list. The ADSP-Lancet AD genes list includes 20 AD genes with genetic evidence compiled by the ADSP Gene Verification committee (accessed by January 2024; last updated by August 17, 2023), plus 81 well-known AD genes from Andrews et al.^24^, totaling 85 unique AD genes (Supplementary Table S3) of strong evidence.

The Multi-platform AD genes list, including genes aggregated from three sources that may of moderate AD-association evidence: Alzheimer’s Disease Variant Portal (ADVP)^20^, Digsee^25^, and the GWAS Catalog^26^. These databases, accessed in January 2024, collectively providing 3,441 unique AD-associated genes (Supplementary Table S4).

For genes not found in either list, we conducted a manual literature review using PubMed and Google Scholar by searching each gene name in combination with the terms “Alzheimer’s disease,” “AD,” or “dementia”.

### Pathway enrichment analysis with GSEA

Pathway enrichment analysis helps researchers gain mechanistic insight into biological pathways by genome-scale experiments. To investigate pathway-level patterns, we used Gene Set Enrichment Analysis (GSEA)^27^, which tests whether predefined gene sets are non-randomly distributed between two biological conditions.

GSEA was performed on GWAS and IBI-DT results from the discovery cohort. Pre-ranked gene lists (*.rnk) were generated for both methods: GWAS genes were ranked by the highest -log10(*P* value) of their leading variant, while IBI-DT genes were ranked by the maximum α of their variants.

### Neural network prediction of AD and SHAP feature importance

We trained a fully connected neural network (NN) to predict AD status using data from the discovery cohort. Input features included top-ranked variants from IBIDT and GWAS, along with six binary-coded non-genetic factors. The neural network architecture consisted of two hidden layers, each with 256 and 128 neurons, followed by ReLU activation, and a sigmoid-activated output layer for binary classification. Models were trained using the Adam optimizer (learning rate = 1e-5) and binary cross-entropy loss, with mini-batch gradient descent across multiple epochs. Performance was evaluated using Receiver Operating Characteristic Area Under the Curve (ROC AUC) on the test dataset. Feature importance was quantified using average of absolute SHAP^28^ values on the test dataset, which estimate each feature’s contribution to individual predictions based on game-theoretic principles. Fully connected Neural network training and SHAP analyses were conducted in Python (v3.10.0).

### Ethical approval and consent to participate

UK Biobank was approved by the North West Multi-Centre Research Ethics Committee, and UK Biobank data for this study were accessed under approval from the UK Biobank Access Management Committee (Application No. 95030). ADSP data were accessed through NIAGADS controlled-access procedures following approval by the NIAGADS ADRD Data Access Committee (NADAC) and Data Use Committee (DUC). All methods were carried out in accordance with relevant guidelines and regulations and with the Declaration of Helsinki. Informed consent was obtained from all participants and/or their legal guardians by the parent studies.

## Results

### Overview

**Fig. 1:**
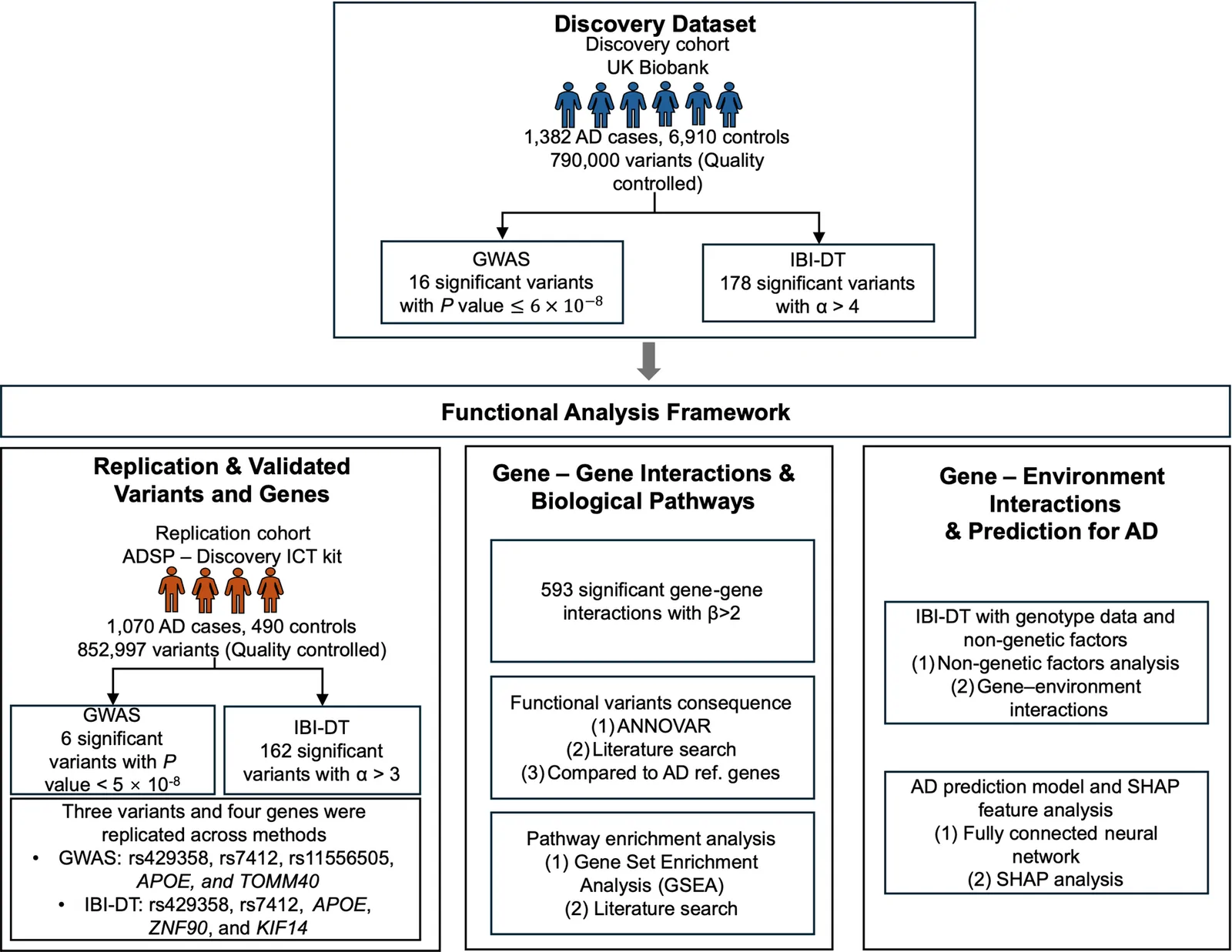
Study design and analysis workflow.

We analyzed 790,000 WES variants from 8,292 unrelated White British individuals in the UK Biobank using both GWAS and IBI-DT to investigate the genetic basis of AD. GWAS identified 16 significant variants, whereas IBI-DT identified 178 using method-specific thresholds. Findings were further evaluated in an independent ADSP-Discovery ICE cohort including 1,560 unrelated individuals and 852,997 variants. Variants were mapped to genes using ANNOVAR and compared with the ADSP-Lancet gene list and a multi-platform AD gene list. We also used IBI-DT to identify gene-gene and gene-environment interactions, performed pathway enrichment analysis, and developed fully connected neural network models with SHAP-based feature interpretation. The overall workflow is shown in Fig. 1.

### IBI-DT prioritized rarer and less correlated variants than GWAS

**Fig. 2:**
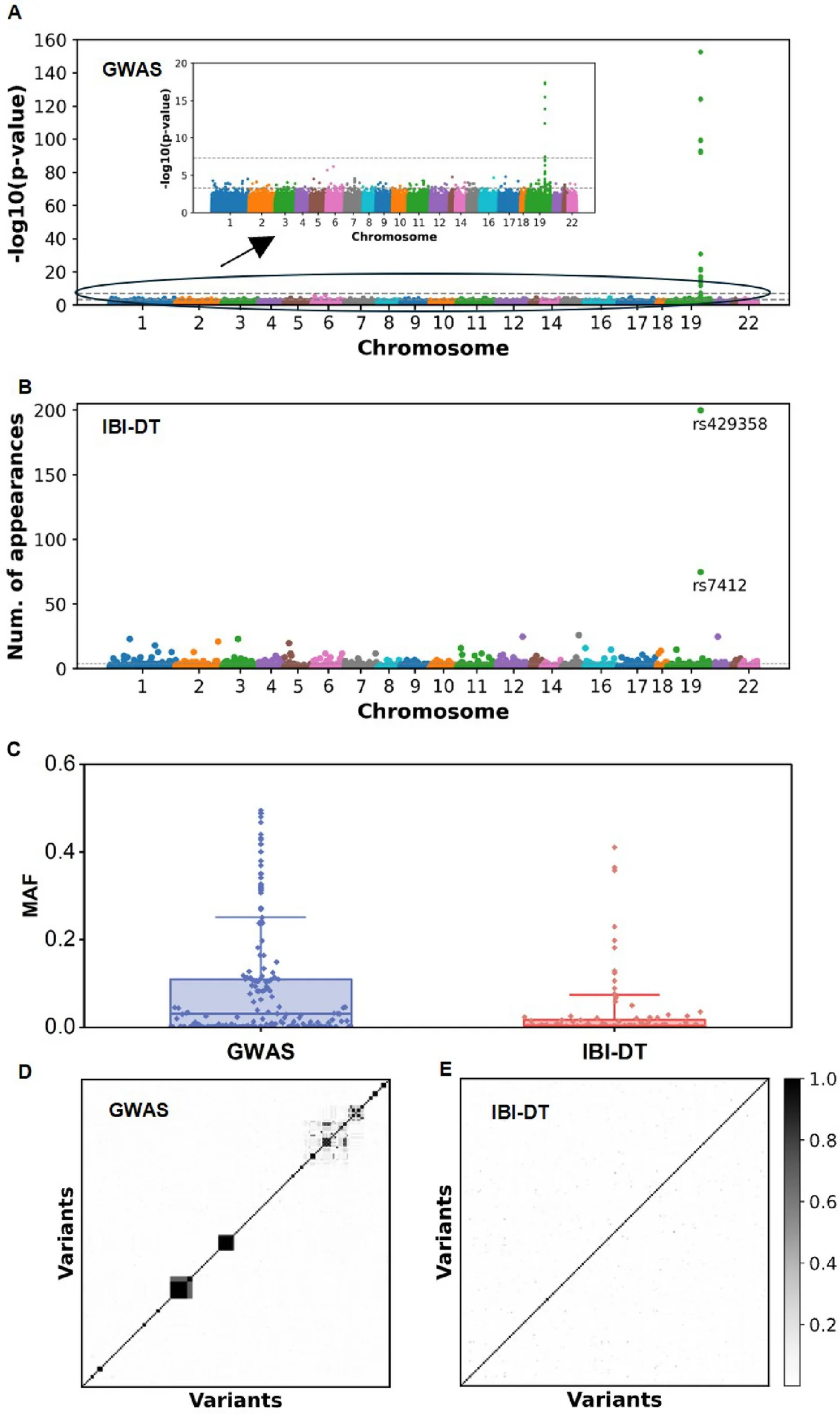
IBI-DT prioritizes more rare and independent variants compared to GWAS. (**A**) Manhattan plot of GWAS results. The X-axis represents chromosome positions, and the Y-axis shows -log10 (*P* value). An inset within panel A magnifies the lower significance region (-log10(*P* value) ≤ 20), providing detail on less extreme but potentially informative signals. Each point represents a variant. The upper dashed line marks the genome-wide significance threshold (*P* value ≤ 5^-8), corresponding to the top 16 variants. The lower dashed line indicates a relaxed threshold (*P* value ≤ 0.0005), which was used to select the top 178 variants for comparison with IBI-DT, matching the number of significant variants identified by IBI-DT. (**B**) Manhattan plot of IBI-DT results. The X-axis represents chromosome positions, and the Y-axis shows number of appearances for each variant for IBI-DT. The IBI-DT threshold (dashed line at 4) identifies 178 significant variants. (**C**) Boxplot of the MAF distribution of 178 significant variants from GWAS and IBI-DT. MAF: minor allele frequency. (**D**, **E**) Heatmap correlation matrices of the top 178 variants identified by GWAS (**D**) and IBI-DT (**E**). Variants are sorted by chromosomal position.

We applied GWAS and IBI-DT to the UKB discovery cohort. In the GWAS analysis, the quantile-quantile plot (Supplementary Fig. 1) showed that most variants closely followed the expected null distribution, with deviation only in the upper tail, supporting the presence of true association signals. A total of 16 variants met the significance threshold (*P* < 0.05/790,000 = 6 × 10^-8), mapping to four genes: *APOE* (lead variant rs429358, *P* = 2 × 10^-153, OR = 4.97, MAF = 0.1814), *TOMM40* (rs11556505, *P* = 2 × 10^-100, OR = 3.65, MAF = 0.1639), *BCAM* (rs28399637, *P* = 6 × 10^-18, OR = 1.68, MAF = 0.3201), and *CBLC* (rs80168591, *P* = 1 × 10^-12, OR = 2.64, MAF = 0.0171) (Fig. 2A). *APOE* was present in both the ADSP-Lancet and multi-platform AD gene lists, whereas *TOMM40*, *BCAM*, and *CBLC* were present in the multi-platform list. The 16 GWAS-significant variants are physically clustered within the broader APOE locus and reduce to fewer LD-clumped regional signals, rather than representing 16 independent loci. Full GWAS results are provided in Supplementary Table S5.

For the IBI-DT analysis, to calibrate the significance threshold for single-variant prioritization, we repeated the random-node control experiment 200 times and generated an empirical null distribution for $\:\alpha\:$. Across the 200 random experiments, the maximum $\:\alpha\:$ value was 2 in 26 experiments, 3 in 172 experiments, and 4 in 2 experiments. The 95th percentile of the null distribution was 3. To use a more conservative criterion, we defined the single-variant significance threshold using the maximum observed null value; therefore, variants with $\:\alpha\:$ >4 were considered significant. Under this threshold, no variant exceeded the cutoff in any of the 200 random-node experiments. In this case, IBI-DT identified 178 significant variants ($\:\alpha\:$ > 4; Fig. 2B). These variants included 82 intronic, 68 exonic, 12 3′ untranslated region (UTR3), 5 5′ untranslated region (UTR5), 4 upstream, 1 intergenic, and 1 non-coding RNA intronic variant. They mapped to 173 genes, including 4 genes in the ADSP-Lancet list and 38 genes in the multi-platform list. Full IBI-DT results are provided in Supplementary Table S6.

To compare allele-frequency patterns between methods, we examined the top 178 variants from each analysis (Fig. 2C). For IBI-DT, these variants were defined by $\:\alpha\:$ > 4. For GWAS, we used a relaxed threshold (*P* < 0.0005) to obtain an equal number of variants. IBI-DT prioritized substantially more rare variants (MAF < = 0.01; 147/178) than GWAS (66/178), and the difference in MAF distributions was significant by two-sample t-test (*P* < 0.0001). At the same time, IBI-DT also identified established common AD signals such as *APOE*. Together, these results suggest that IBI-DT can capture both common and rare AD-associated variants, with particular value for prioritizing low-frequency and rare variants that are often challenging for marginal GWAS. This interpretation is consistent with the later observation that many known, novel, and replicated genes uniquely prioritized by IBI-DT were represented by low-frequency or rare variants. Correlation heatmaps of the top 178 variants (Fig. 2D, E) showed fewer pairwise correlations among IBI-DT-prioritized variants, indicating that IBI-DT tended to select less redundant signals than GWAS.

### Identification of known AD genes in the discovery cohort by both IBI-DT and GWAS

Identified genes were compared with two reference gene sets: the ADSP-Lancet AD gene list and a multi-platform AD gene list described in the Methods. We compared ADSP-Lancet genes represented among the top 178 variants from GWAS and IBI-DT (Table 1). Both methods identified *APOE* and *TREM2*, whereas *ABCA1* and *TPCN1* were identified only by IBI-DT. IBI-DT identified both common and rare AD-related signals. *APOE* was represented by rs429358 and rs7412, with MAFs of 0.181 and 0.076, respectively, whereas *TREM2* was represented by the rare exonic variant rs75932628 (MAF = 0.004). The IBI-DT-only ADSP-Lancet genes were represented by even rarer variants: rs377680134 in *ABCA1* (MAF = 0.0002) and rs148025830 in *TPCN1* (MAF = 0.001). IBI-DT did not prioritize rs769449 because it was highly correlated with rs429358 (Spearman *r* = 0.87), which consistently appeared as the root node across the 200 trees.

**Table 1:** ADSP-Lancet genes for AD identified by GWAS or IBI-DT in the top 178 variants.

rsID	varID (Chr: Position: Ref: Alt)	Nearest gene	Variant type	MAF	*P* value	GWAS rank	IBI-DT rank	Number of appearances
rs429358	19:44908684:T: C	*APOE*	Exonic	0.181	2.00E-153	1	1	200
rs769449	19:44906745:G: A	*APOE*	Exonic	0.149	7.00E-125	2	–	–
rs7412	19:44908822:C: T	*APOE*	Exonic	0.076	4.00E-18	11	2	75
rs75932628	6:41161514:C: T	*TREM2*	Exonic	0.004	6.00E-07	20	22	12
rs377680134	9:104840525:G: A	*ABCA1*	Intronic	0.0002	5.00E-03	1537	71	7
rs148025830	12:113266326:C: T	*TPCN1*	Exonic	0.001	1.00E-02	4565	146	5

**Fig. 3:**
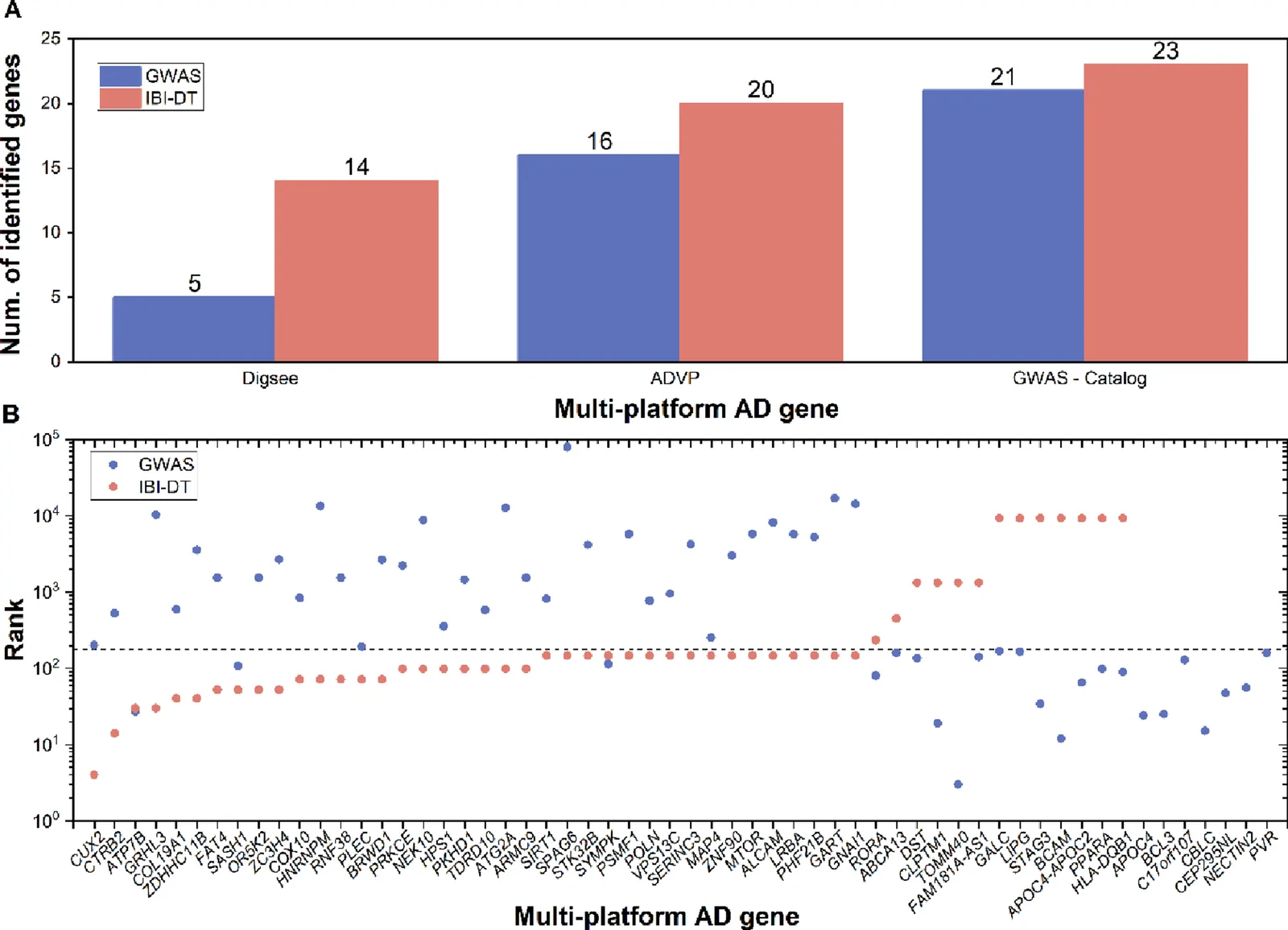
Multi-platform AD genes identified among the top 178 variants by GWAS and IBI-DT. (**A**) Bar plot showing the number of identified known genes across different AD gene databases, comparing GWAS and IBI-DT. (**B**) This figure shows the rank distribution of genes from the multi-platform AD gene list, based on the rank of their lead associated variant among the top 178 variants identified by GWAS (blue) and IBI-DT (red). Each point represents a rank of gene.

We further evaluated these genes against multiple curated AD databases, including DigSee, ADVP, and the GWAS Catalog. Each gene was ranked according to the rank of its lead variant among the top 178 variants from each method. Compared with GWAS, IBI-DT consistently identified more AD-related genes across these databases (Fig. 3A) and uniquely identified many AD genes missed by the parallel GWAS analysis (Fig. 3B). Across the top 178 variants, 58 AD-related genes were identified by either GWAS or IBI-DT. Three genes, *ATP7B*, *SASH1*, and *SYMPK*, were identified by both methods, whereas 35 genes, including *GNAI1*, *HNRNPM*, and *ALCAM*, were identified only by IBI-DT (Fig. 3A, B). Many of the corresponding lead variants for these IBI-DT-only genes were low-frequency or rare, consistent with the allele-frequency pattern observed among the top IBI-DT variants in Fig. 2C. GWAS identified 20 AD genes not prioritized by IBI-DT. Of these, 13 had variants that appeared in at least one of the 200 IBI-DT trees, whereas 7 had no corresponding variants in the trees. Among the 13 variants, three lead variants showed moderate correlation (0.2–0.3) with IBI-DT-significant variants, whereas the remaining ten showed relatively few interactions. These results suggest that IBI-DT can recover established common AD signals while also improving the prioritization of low-frequency or rare AD-related variants that are weakly ranked or missed by marginal GWAS.

### Novel variants and genes uniquely identified by IBI-DT in the discovery cohort

We next evaluated potentially novel genes identified by IBI-DT but not prioritized by GWAS. Among the top 178 IBI-DT variants, many uniquely prioritized signals were exonic or untranslated-region variants, and most of the top novel variants were low-frequency or rare. Specifically, 19 of the top 20 novel genes missed by GWAS had lead variants with MAF < 0.01 (Table 2). We further looked into the variant type and available CADD_phred scores for the 20 IBI-DT-prioritized novel variants listed in Table 2. Among these 20 variants, four were exonic, one was located in the 3′ UTR, one was located in the 5′ UTR, and the remaining 16 were intronic, indicating that most were non-coding variants. CADD_phred scores were available for four variants. rs145202913 annotated to *FERMT1* had the highest CADD_phred score (35), followed by rs138431678 annotated to *CABYR* (12.3), rs139252176 annotated to *SAG* (5.914), and rs142254726 annotated to *MACF1* (0.022). The high CADD_phred score for rs145202913 suggests that this variant may have a potentially deleterious functional effect.

**Table 2:** Top 20 IBI-DT novel genes missed by GWAS.

Gene	rsID	MAF	IBIDT rank	GWAS rank	α	*P*-value	GWAS lead variant rank	Variant type
ADAMTS7*	rs3825808	0.0009	3	2662	26	7.E-03	2662	Intronic
*FERMT1*	rs145202913	0.0011	4	1252	25	4.E-03	1252	Exonic
C3orf14*	rs529355071	0.0019	6	234	23	7.E-04	234	Intronic
MACF1*	rs142254726	0.0004	6	948	23	3.E-03	948	Intronic
*SAG*	rs139252176	0.0047	8	16,399	21	4.E-02	16,399	Intronic
RICTOR*	rs185440036	0.0087	9	3064	20	8.E-03	1537	Intronic
*CD244*	rs151095158	0.0059	10	4565	18	1.E-02	4565	UTR3
CAPN15*	rs562637268	0.0003	12	252	16	8.E-04	252	Intronic
VAV1*	rs1651879	0.0074	14	17,837	15	4.E-02	5745	Intronic
*CABYR*	rs138431678	0.0020	15	45,104	14	9.E-02	45,104	Exonic
PGBD5*	rs373642462	0.0004	17	589	13	2.E-03	589	Exonic
*TSGA10*	rs761217099	0.0009	17	1442	13	5.E-03	1442	Intronic
FASLG*	rs140595317	0.0009	17	20,978	13	5.E-02	20,978	Exonic
*TWSG1*	rs566704748	0.0039	22	395	12	1.E-03	395	Intronic
*ASB10*	rs191838657	0.0009	22	1120	12	3.E-03	1120	Intronic
ALDH3B1*	rs370455543	0.0012	22	2959	12	8.E-03	1537	Intronic
GTF2H5*	rs376278312	0.0009	22	3534	12	9.E-03	3534	UTR5
*NUP98*	rs201908031	0.0061	26	19,868	11	5.E-02	2950	Intronic
*DERA*	rs184071686	0.0004	26	3218	11	9.E-03	3218	Intronic
*EPN3*	rs76487714	0.1061	26	457,042	11	8.E-01	3534	Intronic

This table presents the top 20 novel genes (gene not listed in ADSP-Lancet genes or multi-platform AD genes list) identified by IBI-DT but missed by GWAS. Genes marked with a star indicate that related literature suggests a potential association with AD.

The 178 variants mapped to 173 genes, of which 130 were not present in either the ADSP-Lancet or multi-platform AD gene lists. We performed a manual literature search using PubMed and Google Scholar. Ten of these 20 genes, including *ADAMTS7*, *C3orf14*, *MACF1*, *RICTOR*, *CAPN15*, *VAV1*, *PGBD5*, *FASLG*, *ALDH3B1*, and *GTF2H5*, had prior literature linking them to AD, dementia, cognitive decline, or related neurodegenerative processes. These findings provide suggestive support for the potential biological relevance of some IBI-DT-prioritized genes that were not captured by the parallel GWAS analysis.

### Replication of AD-associated variants and genes in an independent cohort

In the discovery cohort, 790,000 variants mapped to 21,334 unique genes, whereas 852,997 variants in the replication cohort mapped to 22,151 genes. A total of 220,567 variants and 18,185 genes overlapped between the two cohorts.

In the replication cohort, GWAS identified 6 significant variants (*P* < 0.05/852,997 = 5 × 10^-8), whereas IBI-DT identified 162 significant variants ($\:\alpha\:$ > 3). Table 3 summarizes the replicated variants and genes. Three variants were replicated in the replication cohort: rs429358 and rs7412 in *APOE*, and rs11556505 in *TOMM40*, which has previously been associated with increased AD risk and earlier age at onset^29^.

**Table 3:**
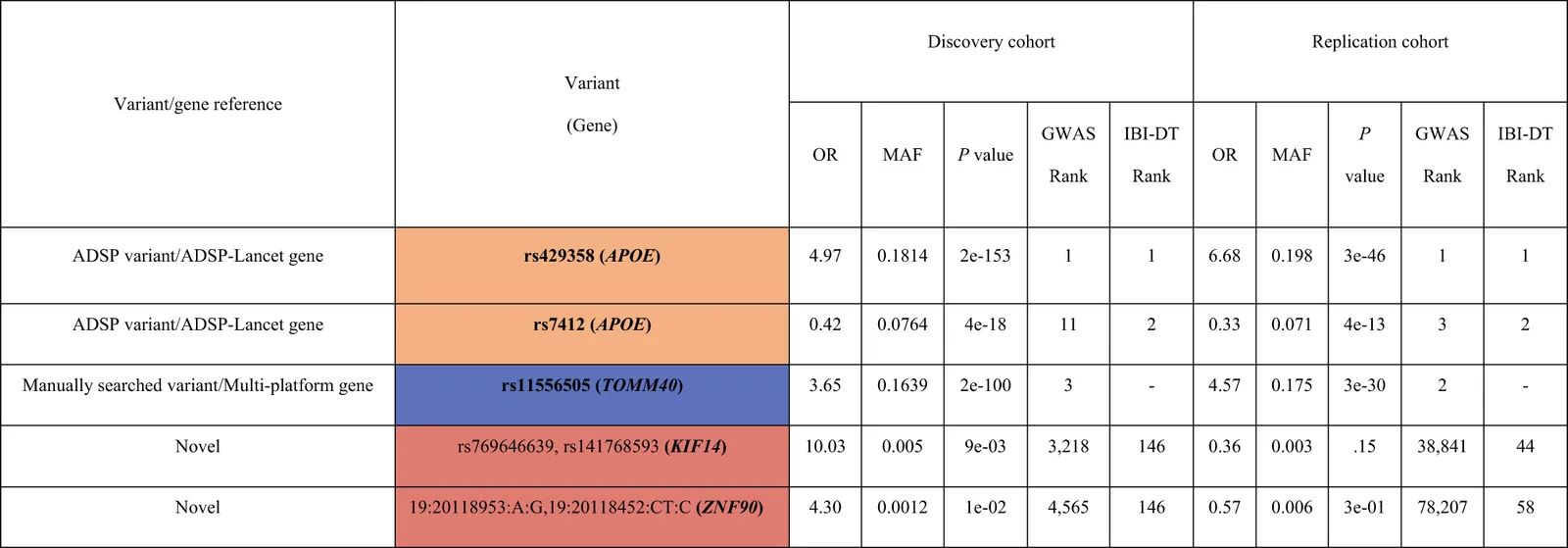
Significant variants and genes identified in both discovery cohort and replication cohort.

This table presents replicated variants and genes; bold formatting indicates those replicated variants/genes. Cell colors highlight replication by different methods: orange indicates replication by both GWAS and IBI-DT, blue indicates GWAS-only replication, and red indicates IBI-DT-only replication. Cells containing two variants represent distinct variants identified in the discovery and replication cohorts for the same gene.

At the gene level, four genes were replicated in the replication cohort. *APOE* was identified by both methods. *TOMM40* was replicated only by GWAS and was not prioritized by IBI-DT, as rs11556505 (mapped to *TOMM40*) has high correlation (Spearman *r* = 0.75) with rs429358 (the root node in IBI-DT tree). This suggests that IBI-DT tends to deprioritize variants that are highly correlated with variants already selected in the tree. In contrast, *KIF14* and *ZNF90* were replicated only by IBI-DT at the gene level, despite ranking much lower in GWAS in both cohorts. In the discovery cohort, *KIF14* and *ZNF90* were represented by rare variant signals with MAFs of 0.005 and 0.0012, respectively, and both ranked 146 by IBI-DT but much lower by GWAS. In the replication cohort, both genes were again prioritized by IBI-DT through different low-frequency lead variants, with MAFs of 0.003 for *KIF14* and 0.006 for *ZNF90*, while their GWAS ranks remained low. These findings support gene-level replication of rare or low-frequency signals by IBI-DT rather than variant-level replication. Because *KIF14* and *ZNF90* were not included in the ADSP-Lancet or multi-platform AD gene lists, we reviewed the literature and found suggestive prior evidence linking KIF14, and more broadly ZNF-family genes, to AD-related mechanisms^30–32^. These genes require further validation in future studies.

### IBI-DT identified significant variants/genes interactions and pathways associated with AD in the discovery cohort

**Fig. 4:**
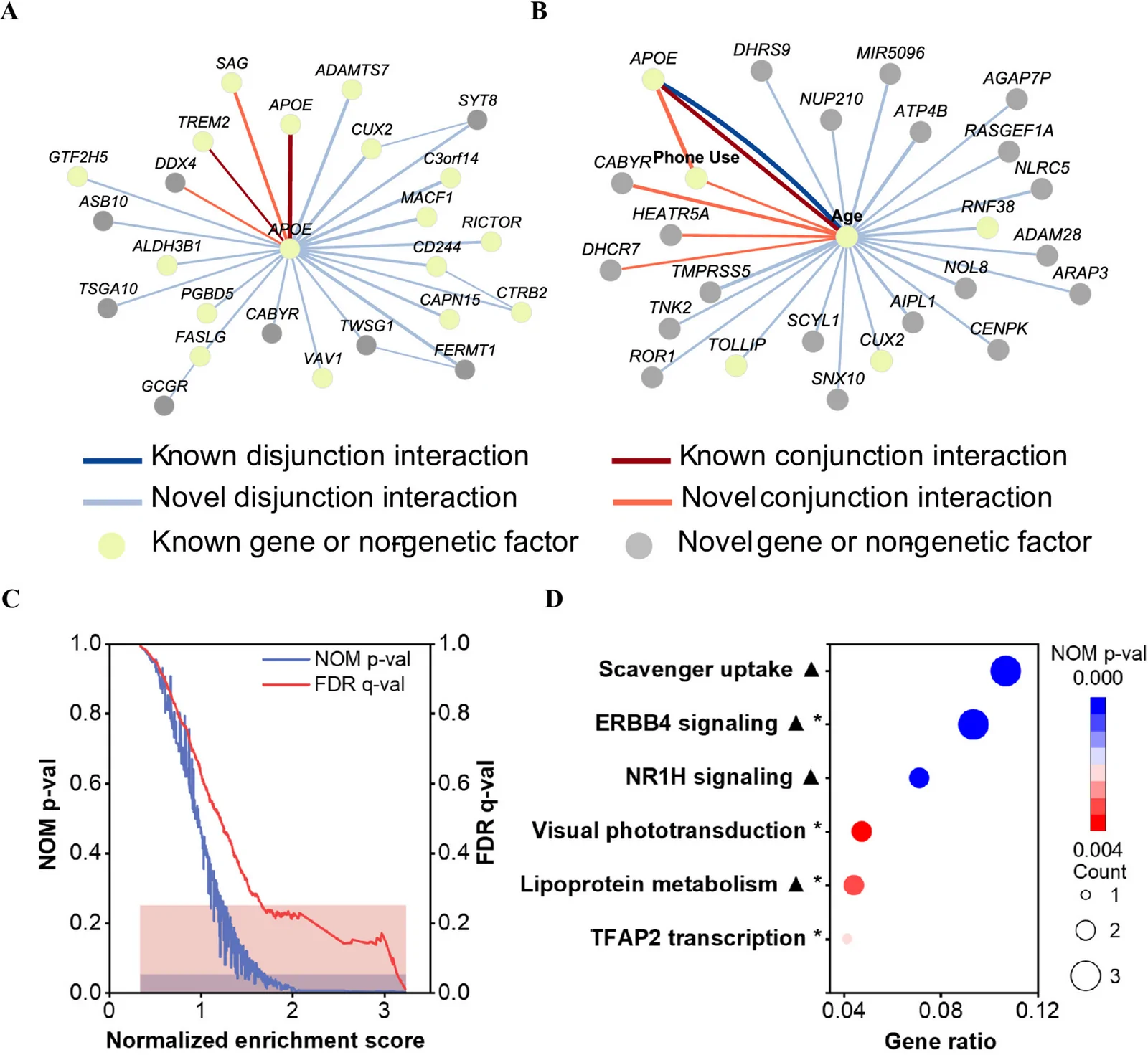
Gene-gene, gene-environment interactions and pathway associated with AD that were identified by IBI-DT from the discovery cohort. (**A**) Top 23 gene-gene interactions and top 4 gene-gene interactions without *APOE* identified by IBI-DT. It includes both known (dark blue) and novel (light blue) disjunctive interactions, as well as known (dark red) and novel (light red) conjunctive interactions. Line thickness corresponds to $\:\beta\:$, with higher $\:\beta\:$ indicating stronger interaction signals and greater association with AD. Known genes (yellow nodes) are defined as those listed in the ADSP-Lancet gene list, the multi-platform gene list, or supported by literature. The variant corresponding to the central *APOE* node is rs429358, another *APOE* variant involved in the network is rs7412. (**B**) Top 28 gene-environment interactions, with non-genetic factors such as age and phone use (shown in bold) interacting with various genes. Known genes or non-genetic factors (yellow nodes) are defined by presence in curated AD gene lists or supporting literature. (**C**) NES vs. significance for nominal *P* values, blue for FDR q-value and red for NOM p-val based on IBI-DT results. The blue rectangle indicates the FDR levels within 0.25, and the red rectangle indicates the nominal *P* value levels within 0.05. Lines in orange color are previously associated with AD. (**D**) The dot plot of top Reactome pathways based on IBI-DT results, the size of each dot represents the number of core enrichment genes, while the color represents the nominal *P* value. The names with the * symbol represent the pathways associated with AD, while others represent the potentially novel pathways associated with AD. The names marked with the ▲ represent the pathway were both identified by GWAS and IBI-DT. The top pathways include Binding and Uptake of Ligands by Scavenger Receptors (Scavenger uptake), Signaling by ERBB4 (ERBB4 signaling), NR1H2 and NR1H3-mediated signaling (NR1H signaling), Visual phototransduction Plasma lipoprotein assembly, remodeling, and clearance (Lipoprotein metabolism), and TFAP2 family of transcription factors (TFAP2 transcription).

*Conjunction and disjunction gene-gene interaction detected by IBI-DT.* IBI-DT detects SNP-SNP interactions that can be mapped to gene-gene interactions in AD. These interactions can be co-occurring (conjunctive) or mutually exclusive (disjunctive), as defined in the IBI-DT framework. To calibrate the significance threshold for variant-variant interactions detected by IBI-DT, we generated an empirical null distribution for $\:\beta\:$ using the same 200 random-node control experiments. Across the 200 random experiments, the maximum $\:\beta\:$ value was 1 in 197 experiments and 2 in 3 experiments. The 95th percentile of the null distribution was 1. To use a more conservative criterion, we defined the interaction significance threshold using the maximum observed null value; therefore, variant pairs with $\:\beta\:$ > 2 were considered significant. Under this threshold, no variant pair exceeded the cutoff in any of the 200 random-node experiments. Using $\:\beta\:$ statistic to quantify interaction frequency across 200 bootstrapped trees, and a significance threshold of $\:\beta\:$ > 2 derived from the random-node experiment, we identified 616 significant SNP-SNP interactions corresponding to 593 gene-gene interactions, including 183 conjunctive and 410 disjunctive interactions; the top 114 gene-gene interactions with β ≥ 6 are summarized in Supplementary Table S7. The strongest interaction was between rs429358 and rs7412, corresponding to an *APOE*-*APOE* interaction ($\:\beta\:$ = 75). We further examined the top 27 gene-gene interactions with strong signals ($\:\beta\:$ >= 12), including 23 involving *APOE* and 4 not involving *APOE* (Fig. 4A). Among these 27 interactions, *APOE*-*APOE* and *APOE*-*TREM2* have been discussed in prior AD literature, and 18 non-*APOE* genes in this set had previously been linked to AD or dementia. To further evaluate the *APOE*-*TREM2* interaction identified by IBI-DT, we tested the corresponding variant pair in a conventional logistic regression framework. The model included the main effects of *APOE* rs429358 and *TREM2* rs75932628 and their multiplicative interaction term. After conditioning on both variant main effects, the *APOE* × *TREM2* interaction term was not statistically significant (*β* = 0.662, *SE* = 0.598, *P* = 0.268), although both variant main effects remained significant. These results indicate that the *APOE*-*TREM2* signal detected by IBI-DT was not supported as a statistically significant global multiplicative interaction in the regression model. This difference is expected because logistic regression tests a single prespecified interaction term across the full sample, whereas IBI-DT identifies stable subgroup-structured interaction patterns across bootstrapped trees. In addition, all variants corresponding to the top 20 IBI-DT novel genes missed by GWAS (Table 2) interacted with the *APOE* variants rs429358 or rs7412, with nearly all such pairs ranking within the top 26 of the 616 significant SNP-SNP interactions.

*Gene-environment interactions detected by IBI-DT*. We next applied IBI-DT to genotype data together with six non-genetic factors selected from the UKB dataset based on availability and prior relevance to AD or cognitive decline: age, insomnia, mobile phone use, education level, Townsend deprivation index, and time spent watching TV. Single-factor analysis ($\:\alpha\:$ > 3) identified age, length of mobile phone use, and educational qualification as significant non-genetic factors, with age showing the highest number of appearances. Using a threshold of $\:\beta\:$ > 2, we identified 736 significant gene-environment interactions. Figure 4B shows the top interactions with $\:\beta\:$ > 12. The most prominent signals involved *APOE*, age, and mobile phone use. In the *APOE*-age interaction, age remained associated with AD in both *APOE* carrier and non-carrier subgroups. *APOE* also showed strong interaction signals with length of mobile phone use.

*Pathways detected by GWAS and IBI-DT.* Pathway enrichment analysis using GSEA and the Reactome database identified 1,043 gene sets in the GWAS analysis, of which 112 met the significance criteria (FDR < 0.25 and nominal *P* < 0.05), and 597 gene sets in the IBI-DT analysis, of which 105 met the same criteria (Fig. 4C). We focused on the top six IBI-DT pathways with normalized enrichment scores greater than 3 (Fig. 4D). Four of these pathways had prior support in the AD literature, and four were also identified by GWAS. Two pathways, visual phototransduction and transcriptional regulation by the AP-2 (TFAP2) family of transcription factors, were uniquely identified by IBI-DT. Two pathways, binding and uptake of ligands by scavenger receptors and NR1H2/NR1H3-mediated signaling, were identified by both methods and may represent additional AD-relevant signals.

### Predictive performance of AD-associated variants and non-genetic risk factors using a neural network

**Fig. 5:**
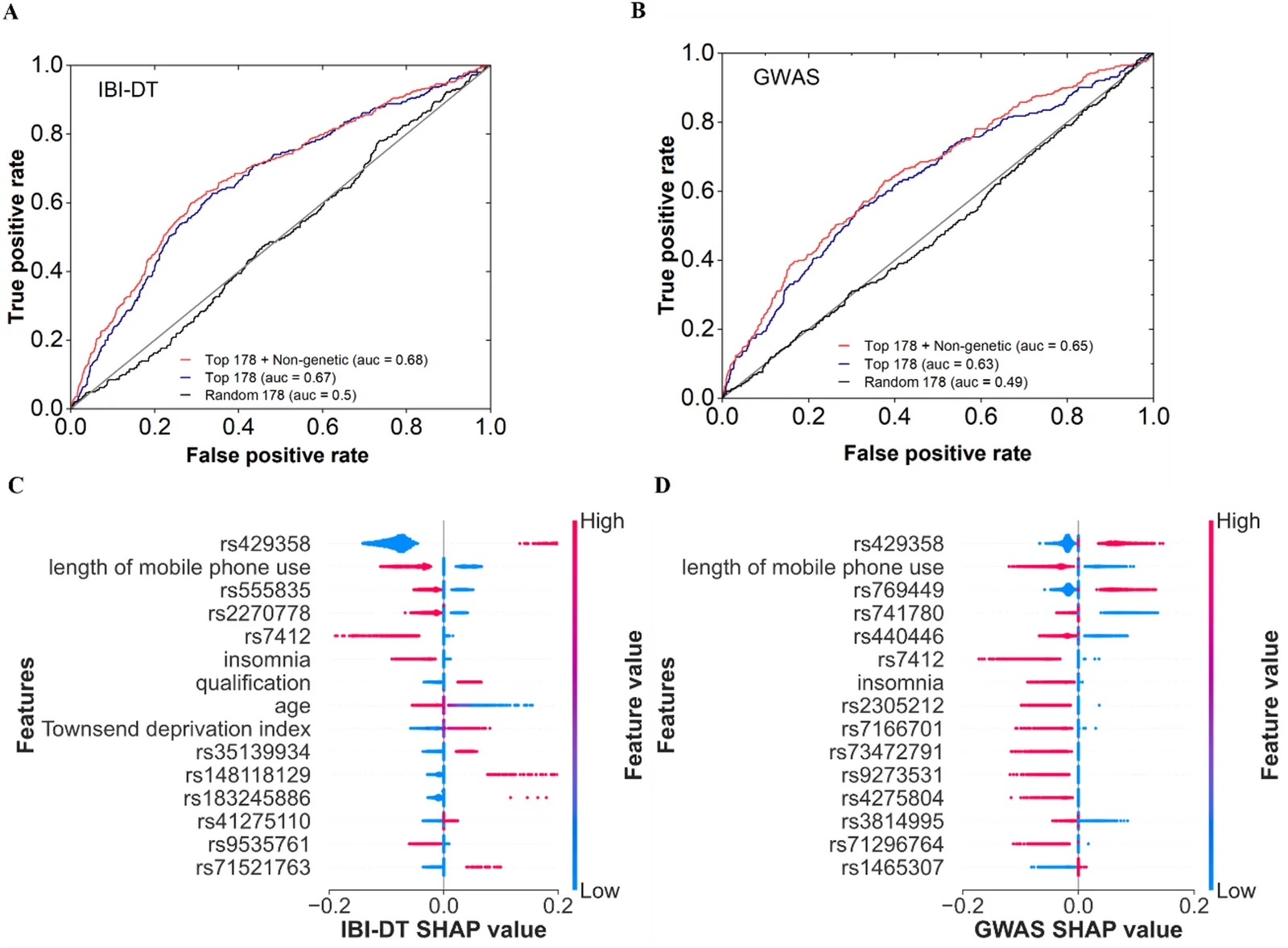
Model performance and SHAP feature importance. (**A**, **B**) ROC curves for neural network models using genetic and non-genetic factors for IBI-DT and GWAS. “Top 178” refers to the top 178 variants selected from IBI-DT or GWAS; “Top 178 + non-genetic” includes these variants plus six additional non-genetic factors: length of mobile phone use, insomnia, qualification, age, Townsend deprivation index and time spent watching. “Random 178” refers to a control set of 178 variants randomly sampled from 790,000 variants. (**C**, **D**) SHAP feature importance for IBI-DT and GWAS NN model using the top 178 variants combined with non-genetic factors. Each dot represents an individual, and colors correspond to raw feature values. SHAP values were computed using the SHAP framework, which quantifies how much each raw feature value shifts an individual’s predicted probability of Alzheimer’s disease relative to an average prediction, which is the average prediction of the model. Positive SHAP values increase the predicted probability of AD (AD case label = 1), while negative values suggest protective effects. Features are ordered by their overall importance. The “High-Low” color bar denotes the raw feature values.

We developed fully connected neural network models to evaluate predictive performance in an unseen subset of the discovery cohort. Figure 5A and B show ROC-AUC curves for models trained using (1) the top 178 variants alone, (2) the top 178 variants plus non-genetic factors, and (3) 178 randomly selected variants as a baseline.

For IBI-DT-prioritized variants (Fig. 5A), the model using the top 178 variants achieved an AUC of 0.67, which increased to 0.68 after adding non-genetic factors. For GWAS-prioritized variants (Fig. 5B), the corresponding AUCs were 0.63 and 0.65. Models trained on 178 randomly selected variants produced near-chance AUCs of 0.50 and 0.49. DeLong’s test showed that the IBI-DT model outperformed the GWAS model when using the top 178 variants alone (AUC 0.67 vs. 0.63, *P* = 0.018).

Feature importance in the neural network models was evaluated using SHAP values (Fig. 5C, D). In both models, rs429358 was the most important predictor, consistent with its rank as the top IBI-DT variant and with prior evidence linking this variant to AD risk^33^. Among the non-genetic factors, length of mobile phone use and insomnia ranked within the top 15 features in both models. Educational qualification, age, and Townsend deprivation index showed higher importance in the model trained on IBI-DT-prioritized variants, suggesting that this variant set may capture additional non-linear or interaction-related patterns involving these factors.

SHAP directionality suggested that longer mobile phone use and educational qualification were associated with lower predicted AD risk, whereas higher Townsend deprivation index was associated with higher predicted risk. Insomnia showed negative SHAP contributions in this dataset. These findings should be interpreted cautiously because SHAP values reflect model behavior within this dataset rather than causal effects.

## Discussion

In this study, we applied both GWAS and IBI-DT to WES data from two independent cohorts, the UK Biobank discovery cohort and the ADSP-Discovery ICE replication cohort, to investigate the genetic architecture of AD. Overall, our findings support IBI-DT as a complementary approach to GWAS. In addition to recovering major established AD signals, including common APOE variants, IBI-DT prioritized additional low-frequency and rare variants that were weakly ranked or missed by GWAS, identified additional genes with suggestive prior biological support that were not highlighted by GWAS, and detected high-order gene-gene and gene-environment interactions that are difficult to capture with conventional association analysis.

A major strength of this study is that IBI-DT recovered known AD-related genes while also identifying additional biologically supported genes beyond the strongest GWAS signals. In the discovery cohort, both methods identified established AD loci such as *APOE* and *TREM2*^33–37^, supporting the validity of the overall analytical framework. Notably, IBI-DT uniquely identified *ABCA1* and *TPCN1*, both of which have prior support in AD^38,39^. *ABCA1* has been implicated in ApoE lipidation, cholesterol transport, and amyloid-related processes, and reduced *ABCA1* expression or activity has been linked to AD pathogenesis, whereas pharmacologic activation of *ABCA1*-related pathways has been proposed as a potential therapeutic strategy^38–40^. *TPCN1* has also been reported in a recent AD GWAS study^12,41^. The corresponding lead variants, rs377680134 in *ABCA1* (MAF = 0.0002) and rs148025830 in *TPCN1* (MAF = 0.001), were both rare variants. This observation is consistent with the minor allele frequency distribution of the prioritized variants, whereby IBI-DT showed a stronger enrichment of rare variants among the top-ranked signals than GWAS. These findings suggest that IBI-DT may help prioritize AD-relevant genes beyond the most prominent marginal GWAS signals, particularly by better capturing rare variants. IBI-DT also highlighted a large set of potentially novel genes. Among the top novel genes uniquely prioritized by IBI-DT, 10 of the top 20 had prior literature support related to AD or dementia, including *ADAMTS*, *C3orf14*, *MACF1*, *RICTOR*, *CAPN15*, *VAV1*, *PGBD5*, *FASLG*, *ALDH3B1*, and *GTF2H5*^42–51^. These genes span several processes relevant to neurodegeneration, including cytoskeletal regulation, immune and inflammatory signaling, transcriptional control, neuronal survival, and cognitive decline. For example, *MACF1* has been linked to cytoskeletal disruption relevant to AD^49^, *CAPN15* has been implicated in brain development and adult brain function^50^, *VAV1* has been connected to neuronal apoptosis and cognitive function in AD models^46^, and *ALDH3B1* has been associated with small-vessel injury in mild cognitive impairment^44^. Although these observations do not establish causal roles, they support the biological plausibility of the novel genes prioritized by IBI-DT and suggest that this method may capture additional candidate components of AD biology not emphasized by GWAS alone.

The replication analysis further strengthens this interpretation. Three variants (rs429358, rs7412, and rs11556505) and four genes (*APOE*, *TOMM40*, *KIF14*, and *ZNF90*) were supported across the discovery and replication cohorts by either GWAS or IBI-DT. The overall replication rate was modest, likely in part because only a limited fraction of the top-ranked discovery variants were represented in the replication dataset. Even under this constraint, *KIF14* and *ZNF90*, represented by rare variants, were replicated only by IBI-DT, while ranking very low in GWAS in both cohorts. Prior literature provides suggestive support for the relevance of these genes. *KIF14* has been reported to move through tau-occupied regions on microtubules^31^ and has been implicated in altered cell cycle-related processes in female AD neurons^32^. Although *ZNF90* itself has not been directly studied in AD, other zinc-finger family genes have been linked to tau pathology, amyloid processing, and glial regulation^30^. Together, these findings suggest that IBI-DT may help prioritize biologically plausible and potentially novel AD-related genes that are not well captured by conventional association testing.

Another important contribution of this study is the identification of interaction structure. IBI-DT detected significant SNP-SNP and gene-gene interactions, including the *APOE*(rs429358)-*APOE*(rs7412) interaction and the *APOE*-*TREM2* interaction, both of which are supported by prior AD literature^34,37^. Although the *APOE*-*TREM2* interaction was not captured as a significant global multiplicative interaction in logistic regression after adjustment for main effects, this result does not contradict the IBI-DT finding. The non-significant *APOE* × *TREM2* regression interaction may partly reflect the limited number of joint genotype carriers, given the rarity of *TREM2* rs75932628. In addition, the strong main effect of *APOE* rs429358 may absorb much of the disease-risk difference, leaving the interaction term to capture only residual deviation beyond both main effects. In contrast, IBI-DT evaluates whether variants define stable subgroup-structured patterns across bootstrapped trees and may therefore capture context-dependent variant relationships that are not well represented by a single global multiplicative interaction coefficient across the full sample. These findings suggest that IBI-DT may be able to recover known biology while also nominating additional candidate interactions for future investigation. Because AD is unlikely to be driven solely by independent marginal effects, the ability to efficiently identify structured higher-order interaction patterns may provide an important complement to standard GWAS and computationally intensive pairwise interaction analyses.

We also investigated gene-environment interactions by incorporating six non-genetic factors into the IBI-DT framework. Significant interactions involved *APOE*, age, educational qualification, and mobile phone use. Age was the most prominent non-genetic factor, consistent with its well-established role in AD risk. The interaction involving length of mobile phone use should be interpreted cautiously. Prior epidemiologic studies reported that long-term cell phone users had a lower risk of hospitalization due to AD and vascular dementia^52^ and that regular mobile phone use was associated with lower risk of all-cause dementia among older adults^53^. However, such associations are difficult to interpret mechanistically and may reflect behavioral, socioeconomic, or other unmeasured correlates rather than a direct protective effect. Therefore, we view this finding as hypothesis-generating and in need of independent validation.

The pathway results provide additional biological context for the IBI-DT-prioritized signals. Several enriched pathways identified by IBI-DT were shared with GWAS, whereas others were uniquely highlighted by IBI-DT. Among these, pathways related to visual phototransduction and transcriptional regulation by the AP-2 (TFAP2) family have some prior links to dementia-related biology^54,55^. In addition, pathways involving scavenger receptor biology and NR1H2/NR1H3-mediated signaling are biologically plausible in the context of AD, given evidence that scavenger receptors expressed on microglia act as receptors for amyloid-β^56^. These pathway-level findings suggest that IBI-DT can recover coherent biological signals beyond single-gene and interaction findings.

Our neural network analyses provide an additional, line of evidence that IBI-DT-prioritized variant set may capture information relevant to AD prediction. Although prediction was not the primary aim of this work, models trained on IBI-DT-prioritized variants showed better discrimination than models trained on GWAS-prioritized variants, and SHAP analysis identified rs429358 as the most important predictor in both frameworks, consistent with the central role of *APOE* in AD^33–35,37^. Together, these findings suggest that the signals prioritized by IBI-DT are not only statistically meaningful but also capture information relevant to downstream prediction.

Several limitations should be noted. First, both cohorts were restricted to individuals of European ancestry, which limits generalizability to other populations. Second, the replication cohort was relatively small, reducing power, especially for rare variants and interaction-related signals. Third, the current implementation of IBI-DT does not explicitly adjust for covariates or kinship structure. We attempted to reduce confounding by restricting analyses to unrelated individuals and by applying an age- and sex-matched nested case-control design in the discovery cohort, but residual confounding remains possible, particularly in the replication cohort. Future extensions of IBI-DT should incorporate covariate adjustment and kinship-aware modeling more directly, either through residualization or integration with mixed-model frameworks. In addition, benchmarking IBI-DT against regression-based interaction models, mixed-model association tests, and rare-variant burden methods will be important for clarifying the settings in which IBI-DT provides the greatest added value. Fourthly, because LD can complicate SNP-level interpretation and biological support is generally stronger at the gene level, our downstream interpretation focused primarily on mapped genes and pathways rather than assigning functional importance to each significant variant. Finally, IBI-DT may miss some strong marginal-effect variants detected by GWAS. Because IBI-DT recursively selects splitting variants using Bayesian marginal likelihood, variants that are highly correlated with a selected lead variant may be deprioritized if they contribute overlapping information to the tree structure, as was observed for rs11556505 in *TOMM40*. Likewise, variants that show fewer interactions with other variants or contribute less to the interaction structure captured by the bootstrapped tree ensemble may not be prioritized, as was mentioned in the results section.

In conclusion, our results suggest that IBI-DT complements GWAS in the analysis of AD WES data by recovering established common AD signals while also detecting additional low-frequency, rare, less correlated, and interaction-related signals. In addition to recovering known AD genes, IBI-DT highlighted additional candidate genes, including *KIF14* and *ZNF90*, and identified gene-gene, gene-environment, and pathway-level signals that require further evaluation to better understand AD genetic architecture. Overall, these findings support further evaluation of IBI-DT as a complementary strategy for discovering AD-associated loci and interaction patterns and nominate several genes and pathways for future validation and mechanistic study.

## Supplementary Information

Supplementary Material 1

Supplementary Material 2

## Data Availability

The UK Biobank data used in this study are available upon application to the UK Biobank resource (https://www.ukbiobank.ac.uk/) under approved Project 95030. The Alzheimer’s Disease Sequencing Project (ADSP) data are available through the ADSP Data Coordination Center (https://adsp.niagads.org/) under controlled access.
