# Supplementary material for: Novel Alzheimer’s disease-associated variants and genetic interactions identified from UK biobank whole-exome sequencing data using IBI-DT
Source: Sci Rep. 2026 Jun 26;16:29518. doi: 10.1038/s41598-026-58159-w (PMC13597298; doi:10.1038/s41598-026-58159-w)
Supplement: Supplementary file 1 [file 41598_2026_58159_MOESM1_ESM.docx]

**Supplemental Figure 1. The quantile-quantile (QQ) plot of GWAS P-values for AD in UKB.**
